# Hospital Contracts and Prices

**Published:** 1910-06-18

**Authors:** 


### HOSPITAL CONTRACTS AND PRICES.

### SURGICAL DRESSINGS.

A correspondent writes: "In your number of The Hospital for May 7 there is an article under the 'Special Departmental Article' on 'Surgical Dressings.' I find that the prices quoted in this article are a good deal lower than what we are at present paying for surgical dressings. I shall be very much obliged if you could let me know the address of the firm which supplies the dressings at the prices mentioned by the writer of the article."

*** If you will furnish us with a list of the dressings you are in the habit of purchasing, and the approximate quantity of each ordered at one time, we shall be glad to advise you where to apply for qualities and samples corresponding with those referred to in the article you indicate.—Ed. Contracts Dept., The Hospital.

